# Biodistribution Analysis of an Anti-EGFR Antibody in the Rat Brain: Validation of CSF Microcirculation as a Viable Pathway to Circumvent the Blood-Brain Barrier for Drug Delivery

**DOI:** 10.3390/pharmaceutics14071441

**Published:** 2022-07-12

**Authors:** Ghazal Naseri Kouzehgarani, Pankaj Kumar, Susan E. Bolin, Edward B. Reilly, Didier R. Lefebvre

**Affiliations:** AbbVie Inc., 1 N Waukegan Road, North Chicago, IL 60064, USA; ghazal.naserikouzehgarani@abbvie.com (G.N.K.); pankaj.kumar2@abbvie.com (P.K.); susan.bolin@abbvie.com (S.E.B.); ed.reilly@abbvie.com (E.B.R.)

**Keywords:** blood-brain barrier, brain targeted therapy, drug delivery, cerebrospinal fluid microcirculation, brain biodistribution

## Abstract

Cerebrospinal fluid (CSF) microcirculation refers to CSF flow through brain or spinal parenchyma. CSF enters the tissue along the perivascular spaces of the penetrating arteries where it mixes with the interstitial fluid circulating through the extracellular space. The potential of harnessing CSF microcirculation for drug delivery to deep areas of the brain remains an area of controversy. This paper sheds additional light on this debate by showing that ABT-806, an EGFR-specific humanized IgG1 monoclonal antibody (mAb), reaches both the cortical and the deep subcortical layers of the rat brain following intra-cisterna magna (ICM) injection. This is significant because the molecular weight of this mAb (150 kDa) is highest among proteins reported to have penetrated deeply into the brain via the CSF route. This finding further confirms the potential of CSF circulation as a drug delivery system for a large subset of molecules offering promise for the treatment of various brain diseases with poor distribution across the blood-brain barrier (BBB). ABT-806 is the parent antibody of ABT-414, an antibody-drug conjugate (ADC) developed to engage EGFR-overexpressing glioblastoma (GBM) tumor cells. To pave the way for future efficacy studies for the treatment of GBM with an intra-CSF administered ADC consisting of a conjugate of ABT-806 (or of one of its close analogs), we verified in vivo the binding of ABT-414 to GBM tumor cells implanted in the cisterna magna and collected toxicity data from both the central nervous system (CNS) and peripheral tissues. The current study supports further exploration of harnessing CSF microcirculation as an alternative to systemic delivery to achieve higher brain tissue exposure, while reducing previously reported ocular toxicity with ABT-414.

## 1. Introduction

Drug delivery via cerebrospinal fluid (CSF) circulation is a pathway allowing the distribution of therapeutics, either small molecules or large biologics, in brain and spinal tissue. This pathway has drawn renewed attention since the recent discovery of the brain-wide perivascular pathway for CSF and interstitial fluid exchange system [1]. For decades, CSF circulation was thought to be limited to flow through the brain ventricles and into the subarachnoid space (SAS) around both the brain and spinal tissue, only exposing contiguous tissues. Now, it is recognized that the CSF also flows through the parenchyma via a mechanism referred to as the CSF microcirculation [2]. The recent literature reported data indicating that this intraparenchymal flow can be harnessed to transport therapeutic molecules to deep regions of the brain [2].

Many biologics with great promise for the treatment of neurodegenerative diseases or tumors have poor brain uptake due to their low permeation across the blood-brain barrier (BBB) following systemic delivery [3,4]. Furthermore, their size is thought to slow transport through brain tissues [5,6,7]. This includes antibody-drug conjugates (ADCs) targeting epidermal growth factor receptors (EGFRs) expressed on glioblastoma (GBM) tumors [8]. As part of an ongoing effort to overcome the biodistribution challenge of tumor-treating ADCs, we report data verifying that CSF microcirculation indeed enables the brain-wide distribution of an IgG-sized antibody. Our primarily tool compound, ABT-806, is a humanized monoclonal parent antibody (mAb) of Depatuxizumab Mafodotin (Depatux-M, also known as ABT-414). Depatux-M is an antibody-drug conjugate in which the parent antibody is conjugated to a potent antimicrotubule agent, monomethyl auristatin F (MMAF). The antibody selectively binds to a unique conformation of human EGFR that is exposed due to EGFR overexpression, gene amplification, or a mutant form of EGFR with deletions of exons 2 through 7 (EGFRvlll) [9,10].

Intellance-I, a previous phase 3 clinical trial using systemic delivery of ABT-414 in GBM patients, did not provide increased survival benefits. This randomized, placebo-controlled study was conducted with intravenous (IV) administration of the ADC in newly diagnosed GBM patients [11]. According to several authors, the BBBs in these patients were not compromised enough to allow for sufficient crossing of the large-sized therapeutic [8,12]. This setback prompted us to explore whether bypassing the BBB via the CSF microcirculation pathway could lead to improved brain exposure, thereby potentially reviving interest in the use of ADCs for the treatment of EGFR^+^ GBM tumors.

Here, we report a time course study of ABT-806 biodistribution in the brain parenchyma showing vastly greater and deeper penetration of the antibody when it was administered via the CSF vs. via the IV route. Our toxicology study indicates no toxicity in the brain or the eye, as opposed to the previously reported ocular toxicity of systemically administered ABT-414 [13,14], further suggesting the potential of the CSF microcirculation as a drug delivery pathway. Lastly, we verify that ABT-414 injected into the rat cisterna magna binds to an EGFR^+^ GBM implant. Although the cisterna magna obviously differs from the location of orthotopic GBM xenografts, it provides a convenient location to verify and optimize the tumor penetration of ABT-414 for future efficacy studies in brain tissues. 

## 2. Materials and Methods

### 2.1. Animals

Male Sprague Dawley rats were purchased from Charles River Laboratories (Wilmington, MA, USA). A total of 57 rats, 8–12-weeks of age at 300–400 g body weight, were used for intra-CSF administration (*n* = 18), IV dosing (*n* = 18), tumor inoculation (*n* = 12) and toxicology study (*n* = 9). Rats were socially housed prior to the study in an enriched environment under a 12:12 h light/dark cycle. Diet and sterile tap water were provided ad libitum. All animal studies were reviewed and approved by AbbVie’s Institutional Animal Care and Use Committee (IACUC). Animal studies were conducted by an Association for Assessment and Accreditation of Laboratory Animal Care International (AAALAC) accredited program and veterinary care was available to ensure appropriate animal care.

### 2.2. Intra-Cisterna Magna (ICM) Cannulation Surgery

Two types of cannulation procedures were performed: acute and chronic. Acute cannulation was used in non-survival surgeries where the test articles were injected immediately after catheter implantation and animals were not subsequently recovered. Chronic cannulation was performed in survival surgeries where the cannula was fixed in place for at least 4 h for a single injection and up to 1 month for weekly injections and the animals were allowed to recover from anesthesia. The surgical technique was the same in both acute and chronic cannulation, as described below, and was adapted from Xavier et al. [15]. All injections were performed under anesthesia. IACUC guidelines were followed for both procedures.

Animals were anesthetized in a 3–4% isoflurane induction chamber. Once they achieved a surgical level of anesthesia, as confirmed by the lack of toe pinch reflexes, the surgical area was clipped of hair and prepared with a surgical disinfectant (povidone-iodine or equivalent) followed by 70% alcohol. Surgical preparation was performed in a station separate from the surgery. The surgeon wore sterile gloves and scrubs. Surgical instruments and materials were sterilized prior to use with the first rat. Between rats, instruments were sterilized by immersion in a hot bead sterilizer for 10–15 s and allowed to cool before being used on another animal.

Animals were fixed in the stereotaxic frame using ear bars and a nose cone to maintain isoflurane anesthesia at 2% during the surgery. Ophthalmic ointment was applied to avoid drying of the eyes. The animal head was tilted at an angle of 120° to the body exposing the protruding occipital crest on the back of the skull which was used as the reference point for making incisions. Using a surgical scalpel, the skin was incised approximately 1 cm along the midline to expose the neck muscles below. Two layers of muscle located underneath the skin were subsequently cut and separated at the midline by the blunt side of the scalpel and were held apart using a retractor. The last muscle layer was pulled apart using either a pair of curved forceps or two cotton tip applicators, taking care to avoid tearing of the dura membrane immediately underneath. The exposed inverted triangular structure in between the cerebellum and the medulla, covered by the translucent dura membrane, was identified as the cisterna magna (CM).

The injection was performed using a cannula made up of a beveled 27-gauge needle attached to a PE20 tubing that was connected to a Hamilton syringe, filled with sterile artificial CSF (ACSF, Tocris Bioscience, Bristol, UK). Using a syringe infusion pump, the tubing was filled with either a fluorescently conjugated ABT-806 or ABT-414 solution, ensuring that an air bubble was introduced to separate the test article and the ACSF. The needle was then inserted into the center of the cisterna magna to a depth of ~1 mm, taking care to not puncture the cerebellum or the medulla. For all surgeries, the cannula was fixed in place by dispensing a mixture of dental cement and cyanoacrylate glue onto the dura membrane. The tubing was cut to an approximate 3–5 cm in length and the end was sealed using a hemostat heated in the hot bead sterilizer. This was done to prevent any CSF leakage and to maintain the intracranial pressure [15].

### 2.3. Acute ICM Cannulation

Immediately after cannula implantation, a dose of 0.3 mg/kg injection was performed with a syringe pump at a rate of 0.8 μL/min to a total volume of 10–15 µL. Post-injection, the test article was allowed to circulate throughout the brain for varying times, i.e., 15 min, 30 min, 45 min, and 60 min, with the cannula remaining in place. At the end of the study and under deep anesthesia, the animal was transferred to a chamber where it was euthanized by CO_2_ overdose followed by formalin perfusion and decapitation. The brain was quickly dissected and fixed by immersion in 10% formalin overnight at 4 °C. The following day, the brain was transferred to a phosphate-buffered saline (PBS) solution before it was sectioned and stained.

### 2.4. Chronic ICM Cannulation

For the 4 h and 24 h timepoints, following 30 min after the injection, the cannula was secured in place as described above and the skin was closed using non-absorbable sutures, wound clips, or tissue adhesive. The animal was recovered from anesthesia and was single housed in a cage to ensure that the cannula was not disturbed. At the respective timepoints, the animal was euthanized, and the brain was collected as explained above.

### 2.5. IV Dosing

The tail vein was used for systemic administration. The rat was made comfortable in a plastic restrainer. The syringe needle was inserted into the tail vein and a dose of either 0.3, 3 or 10 mg/kg of the test article was injected. Pressure was then applied to the site of injection to stop the bleeding. At either 4 h or 24 h post-injection, the animal was euthanized by CO_2_ overdose followed by PBS perfusion and decapitation. The harvested brain was cut in half along the midline. The left hemisphere was fixed in 10% formalin overnight and transferred to PBS the next day for sectioning and immunohistochemistry, whereas the right half was snap frozen in −80 °C for subsequent pharmacokinetic analysis.

### 2.6. Tumor Inoculation and Dosing

The U87Mgde2,7 cell line, an in-house human EGFRvIII mutant GBM tumor cell line, was used for target engagement studies in the cisterna magna in preparation for future experiments in cortical tissues. A bolus of 0.1 × 10^6^ cells in Spinner Modification of Minimum essential Eagle’s medium (SMEM, Sigma-Aldrich, St. Louis, MO, USA) was injected into the CM via the chronic cannulation procedure at a rate of 0.8 µL/min and a total volume of 10 µL. Upon 30 min of injection, the animal was recovered and put back in the home cage. Following 24 h after tumor inoculation, a dose of 0.3 mg/kg of either fluorescently labeled ABT-806, ABT-414, or a 1:1 ratio cocktail of the fluorescent conjugates of ABT-806: ABT-414 was injected under anesthesia through the same implanted cannula. A list of these antibody conjugates is presented in Table 1. At either 30 min, 1 h or 2 h post-injection, the animal was euthanized by CO_2_ overdose followed by formalin perfusion and decapitation, and the brain was collected as described above in the ICM cannulation procedure.

### 2.7. Sectioning, Immunohistochemistry (IHC), and Imaging

Coronal brain slices were prepared at 50-µm thickness using a vibrating blade microtome (Leica, Wetzlar, DE, USA). For the biodistribution studies, the free-floating tissue slices were stained with 4′,6-diamidino-2-phenylindole (DAPI, Thermo Fisher Scientific, Waltham, MA, USA), a nuclear marker, for 4 min followed by PBS washes. Stained sections were then mounted onto glass slides and cover slipped using prolong gold mounting solution (ProLong^®^ Gold Antifade Mountant). For the tumor studies, tissue slices were permeabilized with 0.3% (*v*/*v*) PBS-Triton X-100 (EMD Millipore, Burlington, MA, USA) and blocked with 5% Normal Goat Serum (NGS, Abcam, Cambridge, UK) for 1 h at room temperature. To confirm the identity of the implanted human tumor cells, slices were incubated with human-Lamin A+C antibody (1:250, Abcam) in 0.3% PBS-Triton and 2% NGS for 48 h at 4 °C and subsequently washed with PBS. Sections were then incubated with Alexa Fluor 488 goat anti-rabbit IgG antibody (1:1000, Invitrogen, Waltham, MA, USA) in 0.3% PBS-Triton and 2% NGS for 2 h at room temperature in the dark. DAPI was then added for 4 min, followed by PBS washes, mounting, and cover slipping as explained above [16]. Imaging was performed with the Olympus Fluoview FV1000 confocal laser-scanning microscope.

### 2.8. Pharmacokinetic (PK) Analysis

In the IV administration studies, the snap frozen brain hemispheres were transferred to pre-weighed low binding tubes. Upon thawing on ice, the brain samples were weighed and homogenized using a mixture of protein inhibitor cocktail (Thermo Fisher Scientific, Waltham, MA, USA) and a custom radioimmunoprecipitation assay (RIPA) buffer (Boston Bioproducts, Milford, MA, USA). The homogenates were centrifuged at 13,000 rpms for 10 min at 4 °C and the supernatants were collected and transferred into 2 mL 96-well low binding plates. The plates were sealed and were used for subsequent ligand-binding assay (LBA) analysis.

The LBA analysis was performed with an in-house MSD (Meso Scale Discovery) assay. An MSD assay is an electro-chemiluminescent where the MSD reader measures the intensity of the light generated from the plate once an electric charge is applied [17]. The samples were transferred to streptavidin plates and were blocked by adding blocking buffer for 1 h at room temperature. The samples were then incubated in a 0.1 µg/mL dilution of a biotinylated goat anti-human IgG antibody for 1 h. Prepared standards and quality controls (QCs) were made at 3 different dilutions of 1:5 resulting in 25×, 125× and 625× serial dilutions, and were then added to the plates. Following 1 h at room temperature, the samples were incubated in a 0.1 µg/mL dilution of goat anti-human IgG Sulfo TAG. After 1 h of incubation time, MSD Read Buffer was added, and plates were read using the MSD Sector Imager. The plates were washed after each step. Details of the MSD assay are provided in Table 2.

### 2.9. Toxicology Study

The same procedure as in chronic cannulation surgeries was carried out, except that this time, three weekly injections were performed through the same cannula under anesthesia. The treatment groups received ABT-414 at either 0.3 or 1 mg/kg dose, whereas the control group was administered AB095, a non-targeted isotype control of ABT-806, at a dose of 1 mg/kg. At each injection timepoint, a new catheter made up of a 27-gauge needle tip attached to a PE20 tubing was filled with the test article. The catheter was then quickly connected to the previously implanted cannula in the CM and the solution was injected using a syringe pump. To achieve a final injection volume of 10–15 µL, a volume of 12–17 µL was injected, thereby compensating for the residual solution in the implanted cannula. Post-injection, the test article was allowed to flow through the brain for 30 min before the needle was removed, the CM cannula was sealed, and the animal was recovered [15]. 

### 2.10. Histopathology and Immunohistochemistry

Within 48 h after the last injection, the animal was euthanized by CO_2_ followed by exsanguination. A comprehensive set of tissues was collected and evaluated microscopically. This included the brain and spinal cord with meninges, eyes, heart, lungs, kidneys, liver, peripheral sciatica nerves from right and left legs, spleen, and representative sections of the gastrointestinal tract. To rule out any toxicity related to the surgical procedure of ICM delivery, a trimming scheme recommended for brain sampling in rodents for general toxicity studies was employed. This trimming scheme for brain utilizes 7 coronal sections chosen to contain major functional areas known to be targeted by proven neurotoxicants [18]. The tissue samples were fixed in 10% neutral buffered formalin for 2 days, processed to paraffin blocks, and the sections were stained with hematoxylin and eosin (H&E) for microscopic examination [19].

Vascular Endothelial Growth Factor Receptor 2 (VEGFR2) antibody, an endothelial cell marker, was used for immunohistochemical staining of liver samples from one control animal administered AB095 at 1 mg/kg and one treatment animal administered ABT-414 at 1 mg/kg. Formalin-fixed and paraffin embedded liver sections of 5 mm thickness were stained on BOND RX Automated Research Stainer (Leica Biosystems, Wetzlar, Germany). The relevant details of the IHC assay are provided in the Table 3. The slides were counterstained with hematoxylin, dehydrated in graded alcohol, cleared with xylene, and cover slipped [20].

### 2.11. Data Analysis for IHC Studies

For analysis, an average of 20–25 stained slices per animal (n = 3 animals) per experimental group were imported into ImageJ software (ImageJ, NIH, Bethesda, MD, USA). Regions of interest (ROIs) consisting of brain tissue but excluding the vasculature, were drawn around the puncta in the interstitium, representing the ABT-806 biodistribution in the brain parenchyma. Integrated density and area were calculated for each ROI. To be able to account for staining variances across brains and compare fluorescence intensity across timepoints, background ROIs were selected in areas of the image with no fluorescence. Average fluorescence was calculated for background ROIs and the following formula was used to calculate the fluorescence intensity:

Fluorescence Intensity = Integrated density − (area of selected ROI × mean fluorescence of background)

Statistical difference was calculated by analysis of variance (ANOVA) followed by Tukey’s post-hoc test with significance indicated by *p* < 0.05. The sample size is indicated within the corresponding figure legends. All data are presented as mean ± standard error of mean (SEM).

## 3. Results

**Figure 1:**
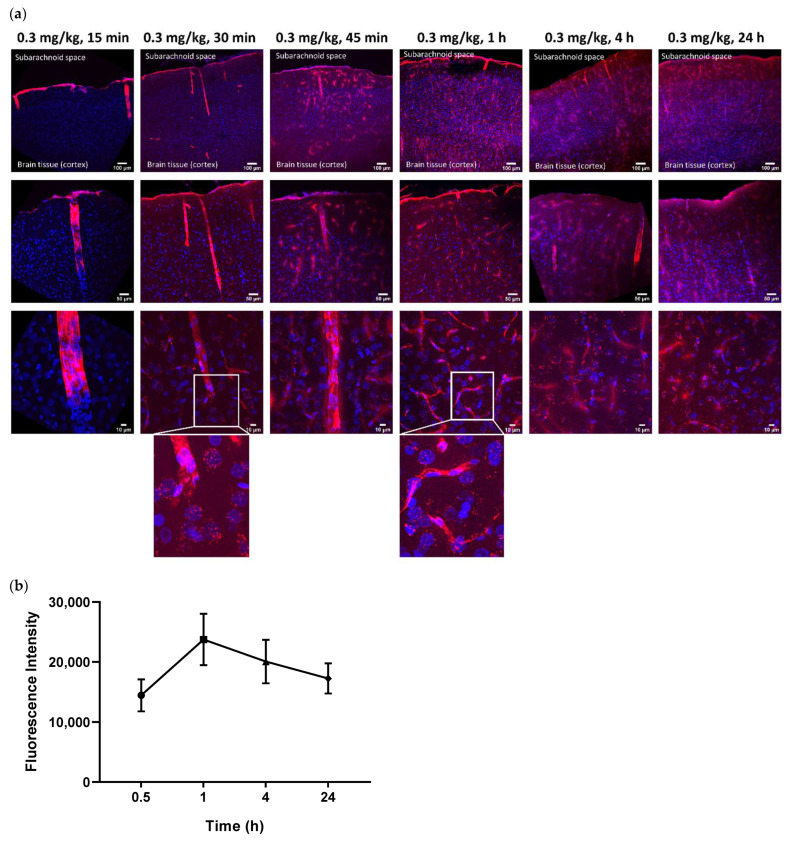
Intra-Cerebrospinal Fluid (CSF) Cortical Biodistribution Data. The parent IgG1 antibody penetrated the brain parenchyma within 30 min of administration into the rat cisterna magna. Representative images. (**a**) Within 15 min post-injection, at a dose of 0.3 mg/kg, intra-CSF administered ABT-806 could only be seen along the periarteriolar spaces with no tissue penetration. After 30 min, the parent antibody started penetrating the brain parenchyma via the periarteriolar spaces. More penetration was observed after 45 min and 60 min of administration with ABT-806 starting to clear out of the parenchyma via the perivenular spaces. The parent antibody continued to enter the parenchyma even after 4 h post-injection. After 24 h, ABT-806 was still present in the tissue. Blue = 4′,6-diamidino-2-phenylindole (DAPI), Red = ABT-806. (**b**) Quantification of the fluorescence intensity in the parenchyma but excluding the vasculature, indicates highest antibody concentrations at 1 h post-injection. 0.5 h: 14,437 ± 2655; 1 h: 23,750 ± 4280; 4 h: 20,063 ± 3635; 24 h: 17,245 ± 2514. *p* > 0.05. *n* = 3 animals per timepoint, average of 20–25 slices per animal per timepoint.

To assess whether an intra-CSF injected antibody of size 150 kDa can penetrate the brain parenchyma, we ran a time course study by injecting a fluorescently conjugated ABT-806 solution at a dose of 0.3 mg/kg into the cisterna magna of three rats per timepoint. Ex vivo tissue slices were evaluated for the extent of antibody penetration into the cortex and were compared across multiple timepoints. We found that the antibody followed the well-established route of CSF flow from the CM to the SAS surrounding the brain tissue, as observed by the continuous red color along the edges of the slices (Figure 1a). Subsequently, the antibody entered the cortex through the perivascular spaces along the large penetrating arteries that branched into arterioles. This observation matched the anatomy of these structures where smooth muscle cells form concentric rings along the arteriole lumen [21,22,23]. The smooth muscle cell morphology is revealed in the bottom panel under 15 min post-injection time, at which timepoint no tissue penetration was observed (Figure 1a).

Within 30 min post-injection, the antibody continued to follow the path of CSF microcirculation and was released into the cortical parenchyma via the periarteriolar spaces, as observed by the red puncta near and around the cell nuclei. ABT-806 binds to a unique epitope on EGFR which is only exposed when EGFR is overexpressed or mutated [9]. Since EGFR in healthy tissue is not commonly found in the overexpressed or mutated conformation, our observation suggests non-specific binding of the antibody to the brain cells and the interstitial space (Figure 1a).

Brain harvest after 45 min and 1 h of ICM administration displayed a higher number of penetrating arteries carrying the antibody into the cortex along their perivascular spaces. Additionally, an increased number of red puncta was observed in the brain parenchyma, suggesting a greater extent of tissue penetration. Concurrently, uptake by the perivenular spaces was observed. This observation was confirmed by the anatomy of venules specified by the stellate shape of the smooth muscle cells with interwoven formations around the lumen of the vessels [21,22,23], noted in the bottom panel under 60 min timepoint (Figure 1a).

Interestingly, after 4 h of ICM injection, the antibody continued to enter the cortex along perivascular spaces and permeated the tissue. Although more ABT-806 was observed to be cleared via the perivenular spaces, the antibody was still present in the brain parenchyma following 24 h post-injection (Figure 1a).

Quantification of the fluorescence intensity in the parenchyma but excluding the vasculature, showed the highest antibody concentration in the cortical parenchyma at 1 h post-injection. It was noteworthy that the mAb concentration in the tissue after 24 h was still higher than that at 30 min following ICM administration (Figure 1b). However, no statistical significance was reached at any timepoint (*p* > 0.05).

**Figure 2:**
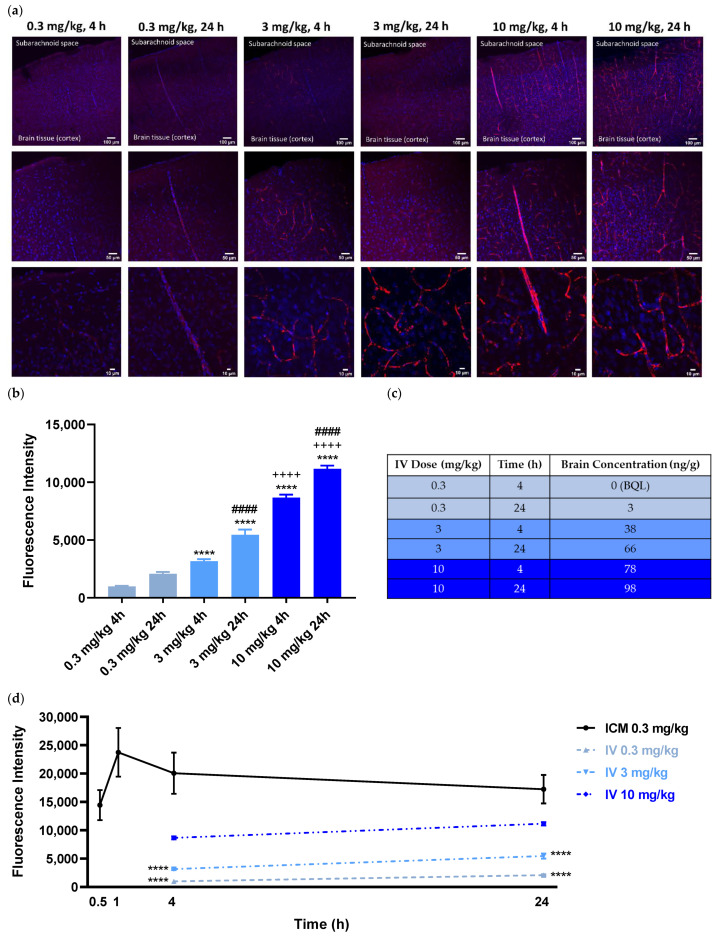
Intravenous (IV) Cortical Biodistribution Data. At equal dose, the tissue penetration of the parent IgG1 antibody administered via the rat tail vein was an order of magnitude lower than when administered intra-CSF. Representative images. (**a**) Upon IV delivery of ABT-806 at 0.3 mg/kg (same dose as administered intra-CSF), the parent antibody was barely detectable in the brain tissue, both within 4 h and 24 h post-injection. Within 4 h of tail vein injection at 3 mg/kg (10-times higher than administered intra-CSF), ABT-806 was observed in trace amounts in the brain tissue. Even at 10 mg/kg, (~30-fold higher than administered intra-CSF), IV-delivered ABT-806 resulted in low penetration in brain tissue, consistent with a poorly permeable blood-brain barrier (BBB). Blue = DAPI, Red = ABT-806. (**b**) Quantification of the fluorescence intensity (vasculature excluded) displays a dose-response relationship which is also time-dependent. 0.3 mg/kg 4 h: 979.1 ± 51; 0.3 mg/kg 24 h: 2085 ± 163; 3 mg/kg 4 h: 3166 ± 180; 3 mg/kg 24 h: 5452 ± 452; 10 mg/kg 4 h: 8663 ± 272; 10 mg/kg 24 h: 11155 ± 292. **** *p* < 0.0001 vs. 0.3 mg/kg at that timepoint, ++++ *p* < 0.0001 vs. 3 mg/kg at that timepoint, #### *p* < 0.0001 vs. same dose at 4 h; One-way ANOVA, Tukey’s post-hoc test. *n* = 3 animals per timepoint, average of 20–25 slices per animal per timepoint. (**c**) The IHC observation and quantification are consistent with the results from the PK analysis of IV-administered brain homogenates that are summarized in the table. BQL = Below Quantification Limit. (**d**) Comparison of the fluorescence intensity between ICM and IV administered brains indicates an order of magnitude higher exposures with intra-CSF vs. systemic administration at 0.3 mg/kg dose. **** *p* < 0.0001 vs. ICM at that timepoint; Two-way ANOVA, Tukey’s post-hoc test.

In a control study aimed to compare the extent of antibody penetration with systemic administration as opposed to intra-CSF, fluorescently labeled ABT-806 was injected into the rat tail vein at three separate doses and the brains were assessed either 4 h or 24 h post-injection in three rats per experimental group. The dose of 3 mg/kg was chosen to match previous experiments showing antitumor activity at this dose after systemic administration. For bracketing purpose, both a lower and a higher dose were added to the experimental design. The 4 h and 24 h timepoints were selected according to a preliminary DMPK simulation suggesting that upon IV administration, brain concentrations peak at ~ 4 h and plateau at ~ 24 h (data not shown). Injection of a dose of 0.3 mg/kg, the same dose as administered in the ICM studies, resulted in very little red fluorescence inside the brain tissue at either 4 h or 24 h. At 3 mg/kg and 10 mg/kg doses, (10- and ~30-fold higher than administered intra-CSF, respectively), only trace amounts of antibody were visible in the brain parenchyma. This observation further confirms the low permeability of the BBB to molecules of large size when administered systemically (Figure 2a).

Quantification of the fluorescence intensity indicated the antibody concentration to be both dose- and time-dependent. The concentration of the fluorescently labeled ABT-806 was lowest at the 0.3 mg/kg dose at 4 h and highest at the 10 mg/kg at 24 h (*p* < 0.0001) (Figure 2b). The results from the PK analysis matched our ex vivo imaging observations. The fluorescently labeled ABT-806 was non-detectable in the brain homogenate within 4 h of IV injection at 0.3 mg/kg, consistent with our finding of trace amounts of antibody in the brain slices. Upon 24 h of administration, the antibody concentration was reported at 3 ng per g of total brain homogenate weight. Higher doses of 3 and 10 mg/kg at 4 h post-injection resulted in greater brain concentrations of 38 and 78 ng/g, respectively. Furthermore, the antibody concentration was reported to increase to 66 and 98 ng/g within 24 h of injection at the two higher doses (Figure 2c).

Comparison of the fluorescence intensity between ICM and IV groups showed an order of magnitude higher antibody concentrations in the brain parenchyma following intra-CSF administration at 0.3 mg/kg. At 4 h and 24 h post ICM injections at a dose of 0.3 mg/kg, fluorescence intensity was significantly higher than that of IV administration at either 0.3 or 3 mg/kg (*p* < 0.0001). Interestingly, following 24 h after IV dosing at 10 mg/kg, the antibody concentration was still lower than that of 30 min post-ICM injection at 0.3 mg/kg (~30-fold lower dose) (Figure 2d).

**Figure 3:**
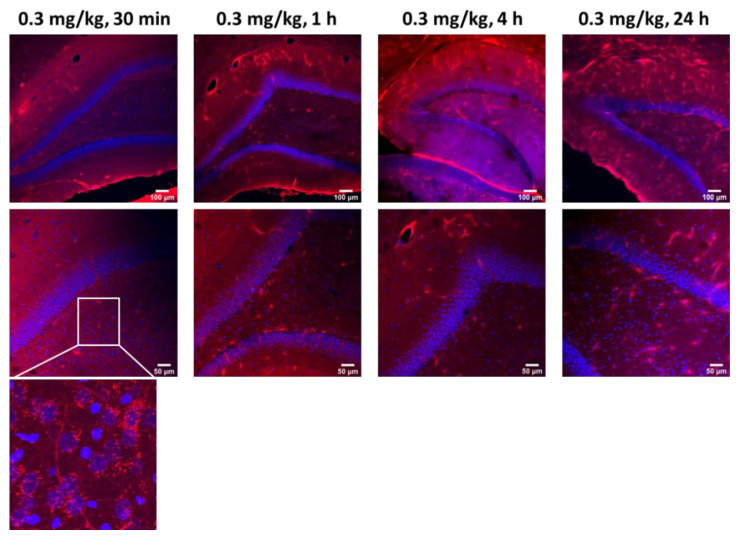
Intra-CSF Subcortical Biodistribution Data. The parent IgG1 antibody penetrated deep into the hippocampal parenchyma within 30 min of administration into the rat cisterna magna. Representative images. Intra-CSF delivery of ABT-806 at a dose of 0.3 mg/kg resulted in the penetration of the antibody into the deeper brain regions, including the hippocampus, as early as 30 min post injection. The antibody was still present in the subcortical regions after 24 h. Blue = DAPI, Red = ABT-806. *n* = 3 animals per timepoint.

To assess the extent of antibody penetration via intra-CSF administration, subcortical brain regions including the hippocampus, were analyzed ex vivo, in three animal brains per timepoint. Within 30 min post-injection, ABT-806 delivered via the cisterna magna at a dose of 0.3 mg/kg had penetrated the dentate gyrus layer of the hippocampus, including its molecular layer, subgranular zone and the hilus. The antibody presence was observed in these layers following 1 h, 4 h and 24 h after injection (Figure 3).

Target engagement studies were performed to evaluate the in vivo binding of ABT-806 and ABT-414 to GBM tumor cells directly implanted in the cisterna magna of three rats per experimental group. The cisterna magna was chosen as a prelude to future experiments to be conducted with orthotopic implants located in the cortical or subcortical area of the parenchyma. The CM was deemed to be an “easy-to-reach” area of the brain to verify target engagement and to study the effect of ADC dilution without introducing too many variables. Efficacy is not reported here. This will be investigated in a follow-up study with orthotopically implanted GBM cells. 

**Figure 4:**
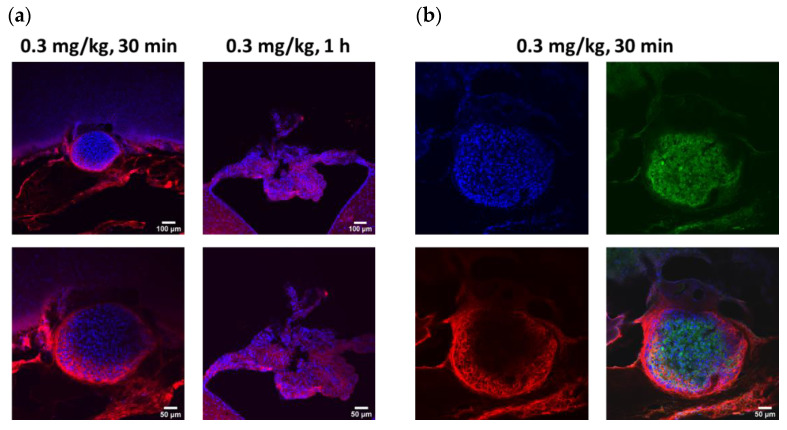

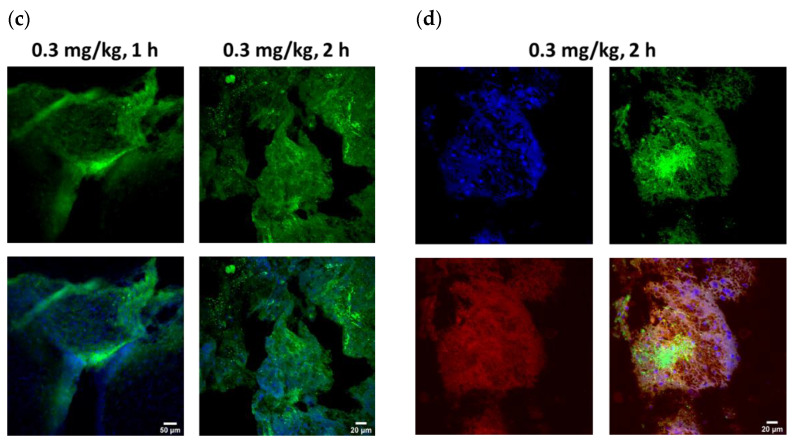
Target Engagement. Intra-CSF injection of U87MGde2,7 tumor cell line followed by next day intra-cisterna magna (ICM) delivery of fluorescently labeled ABT-806 or ABT-414 (FL-ABT-806 or FL-ABT-414) resulted in in vivo binding. Representative images. (**a**) FL-ABT-806 bound to tumor cells after 30 min of intra-CSF delivery. Antibody penetration was limited to the tumor periphery, as evidenced by the red color only on the outer layer of the tumor cluster. The antibody penetrated deeper into the tumor cluster 1 h post-injection, as seen by the red puncta inside. (**b**) To confirm that FL-ABT-806 bound to human tumor cells as opposed to the rat tissue, human-Lamin A + C antibody was used for immunostaining. This antibody colocalized with DAPI only in the tumor cell cluster, thereby confirming the human nature of the cells. Blue = DAPI, Red = FL-ABT-806, Green = Human-Lamin A+C. (**c**) Intra-CSF delivery of FL-ABT-414 resulted in deeper penetration into the tumor cluster 2 h vs. 1 h post-injection, as evidenced by more green puncta inside the tumor. (**d**) ICM administration of a “cocktail” with 1:1 ratio of FL-conjugates of ABT-806: ABT-414 increased penetration of FL-ABT-414 into the tumor cluster core after 2 h of injection, as seen by a higher intensity of green color inside the tumor. Blue = DAPI, Red = FL-ABT-806, Green = FL-ABT-414. *n* = 3 animals per treatment group.

The injected tumor cells formed clusters within 24 h of injection and attached to the cell linings of the cisterna magna and the fourth ventricle. Within 30 min of ABT-806 administration, in vivo binding to the outer layer of the tumor cell cluster was observed. This observation suggested penetration to the core might have been hindered by receptor saturation at the tumor periphery. Deeper penetration of the parent antibody was found after 1 h (Figure 4a). To confirm the binding of the antibody to human tumor cells as opposed to rat tissue, human-Lamin A + C antibody, a nuclear envelope marker, was used for immunostaining. This antibody colocalized with DAPI, a nuclear cell marker, only in the tumor cell cluster, which further confirmed the nature of the cell cluster as human (Figure 4b). Additionally, ICM administration of ABT-414 within 24 h of tumor cell implantation in the cisterna magna resulted in in vivo binding. The extent of the ADC penetration into the core of the tumor cluster was greater after 2 h vs. 1 h (Figure 4c). To verify our receptor saturation hypothesis, we injected a 1:1 ratio mixture of ABT-806: ABT-414 through the CM cannula and assessed target engagement 2 h post-injection. We found a higher concentration of ABT-414 within the tumor cluster core, as evidenced by a brighter fluorescence intensity (Figure 4d). This finding was a confirmation that we could indeed increase ADC transport to the center of the tumor cell cluster by utilizing the parent antibody to occupy target GBM receptors at the periphery.

**Figure 5:**
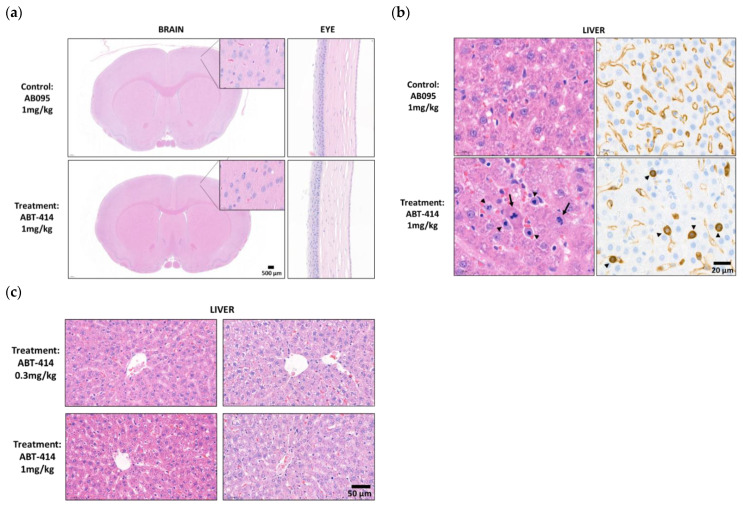
Toxicity Data. No toxicity was found with ICM delivery of ABT-414 in the brain. Systemic exposure was observed in the liver of one rat. Representative images of brain (left), eye (middle), and liver (right) from one control animal administered 1 mg/kg AB095 (upper panel) and one treatment animal administered 1 mg/kg ABT-414 (lower panel). (**a**) There was no toxicity in the brain (inset showing healthy neurons from the cerebral cortex) and eye (cornea). (**b**) In the liver of one animal administered 1 mg/kg ABT-414, there were increased number of mitotic figures and single cell necrosis within hepatocytes (arrows) and mitotically arrested sinusoidal cells (arrow heads) in the animal administered ABT-414 (H&E). Sinusoidal cells showed strong reactivity to Vascular Endothelial Growth Factor Receptor 2 (VEGFR2) immunostaining (arrow heads, lower right corner). In contrast, VEGFR2 staining of control rat liver tissue showed diffuse and continuous positive signal in sinusoidal endothelial cells (SECs) lining the hepatic cords (upper right corner) (IHC). (**c**) There were no microscopic liver changes associated with ICM delivery of a lower dose of ABT-414 at 0.3 mg/kg. The other two rats administered ABT-414 at 1 mg/kg did not show any microscopic findings in the liver (H&E). *n* = 3 animals per treatment group.

The toxicology study indicated no definite microscopic findings directly related to ICM delivery in three rats per experimental group receiving either AB095 (non-specific and payload-free) or ABT-414 (target binding) antibodies. There were no microscopic changes indicative of neurotoxicity in either the brain or the spinal cord such as neuronal necrosis, gliosis, or axonal degeneration (Figure 5a). No inflammation or neuronal necrosis in the neuropil adjacent to the site of catheter placement was observed. Additionally, there were no significant microscopic findings in the eye. Emphasis was placed on evaluating cornea as corneal toxicity has been reported after intravenous administration of ABT-414 in previous nonclinical studies [24] and clinical trials [13] (Figure 5a).

The only ABT-414-related microscopic findings were observed in the liver of a single animal at 1 mg/kg and were characterized by increased numbers of atypical mitotic figures representing metaphase arrest within hepatocytes and sinusoidal cells. The sinusoidal cells were demonstrated to be sinusoidal endothelial cells (SECs) by immunostaining with endothelial cell marker VEGFR2 (Figure 5b). These minimal liver findings were qualitatively consistent with those observed previously with intravenous administration of ABT-414 and were indicative of limited systemic exposure to the drug. However, such microscopic liver observations were not found in either the 0.3 mg/kg treatment group, nor the other two rats receiving the 1 mg/kg dose (Figure 5c).

## 4. Discussion

The current study verifies the feasibility of utilizing intra-CSF delivery to achieve deep brain exposure with high molecular weight biologics. This is consistent with earlier studies from Iliff et al., 2012 and 2013 [1,25,26], Yadav et al., 2017 [27], and Pizzo et al., 2018 [28] and further confirms that CSF microcirculation holds promise to deliver antibodies into deep brain regions. It is noteworthy that the molecular weight, depth of penetration and imaging resolution reported here, i.e., requisites for the delivery of molecules the size of mAbs and ADCs by bypassing the BBB, were all greater than those reported in previously published studies.

In the landmark study of CSF microcirculation discovery, Iliff et al. injected fluorescent tracers of small (759 Da and 3 kDa), intermediate (45 kDa), and large (500 kDa and 2000 kDa) molecular weight into the cisterna magna of rodents. They observed that in contrast to dyes of small or intermediate size that penetrated the interstitium within 30 min of injection, the 500 kDa and 2000 kDa tracers remained confined to the perivascular spaces [1,25,26]. While these studies were significant milestones, the feasibility of achieving similar results with a molecule in the orders of hundreds of kDa molecular weight, such as a 150 kDa antibody, remained unexplored. Additionally, tissue penetration was only reported at the level of the cortex within a 240 µm depth from the surface using in vivo two-photon confocal microscopy [1,26]. In comparison our study reports penetration within mm depths of the cortical surface. The ex vivo fluorescence distribution analysis of the whole brain slices was quantified at a 4× magnification and at 30 min post-injection timepoint only [1]. Our data span quantification up to 24 h following intra-CSF administration and at 64× magnification. 

Yadav et al. performed a 6-week continuous intracerebroventricular (ICV) infusion study in non-human primates with a 56 kDa anti-BACE1 IgG antibody as well as a 150 kDa control IgG antibody. They observed uniform distribution of both antibodies throughout the brain parenchyma including both cortical and subcortical regions, not unlike what we found in our biodistribution findings. The brain concentrations were quantified using ELISA on brain homogenates as well as ex vivo fluorescence microscopy on whole brain slices at a low imaging resolution [27]. Our ex vivo analysis of brain slices increased resolution significantly. This was accomplished by drawing multiple ROIs in each brain slice only around the puncta indicative of mAb penetration while making sure to exclude the perivascular spaces, averaging the fluorescence intensity of those ROIs and correcting against the background in an average of ~70 slices per timepoint. 

Pizzo et al. compared the brain penetration of a 16.8 kDa single-domain antibody (sdAb) to that of a 150 kDa IgG antibody with ICM infusion. Using ex vivo fluorescence imaging and in vivo 3D magnetic resonance imaging (MRI), they observed widespread distribution of both molecules in deep brain regions where the sdAb crossed the perivascular spaces much more easily than the full-size antibody, resulting in a four to seven-fold higher brain exposure as measured by the percentage area with antibody signal within brain slices [28]. Our research builds on Pizzo et al. work by revealing extensive mAb penetration in deep brain areas using a quantification technique with higher resolution in the brain tissue but excluding the perivascular spaces. Using punctate immunostaining as evidence for parenchymal penetration has been previously used with IV administered BBB-permeable single-domain antibodies [29].

Our study demonstrates that an intra-CSF administered antibody follows the recently established path of CSF microcirculation by flowing along the perivascular spaces before distributing itself into the interstitial space of both cortical and subcortical brain regions. Our finding of antibody penetration into the brain tissue was based on the observation of large numbers of puncta near and around the cell nuclei, suggesting non-specific binding. Non-specific binding can result from binding of the antibody to amino acids outside of the target epitope of the antigen. This includes interactions between the antibody, serum proteins and/or endogenous molecules in the tissue that can affect the IHC detection [30]. Non-specific binding can result in high background staining which in turn, can cause the target antigen to optically appear at the incorrect location. However, such artifacts did not perturb our findings, as the antigen of interest, i.e., overexpressed or mutated EGFR, is not found in healthy brain tissues. This gives us confidence that the observed non-specific binding indicates permeation of the antibody into the interstitium.

The dose administered in our ICM administration studies was an order of magnitude lower than that administered systemically in preclinical studies and clinical trials with ABT-414 [10,11,14]. We hypothesized that by bypassing the BBB, ICM injection would give rise to higher brain concentrations of antibodies at lower doses compared to IV administration. In addition to using the same 0.3 mg/kg ICM dose, two higher doses of 3 and 10 mg/kg were selected for IV delivery to facilitate comparison with prior studies with systemic administration [10,11,14].

Quantification of the fluorescence intensity in ICM administered brain slices represents relative concentrations of ABT-806 in the parenchyma. Although semi-quantitative from the standpoint of drug concentration in the parenchyma, the preliminary PK data is remarkable in that the rates of uptake and clearance are quite high, considering that the transport of large molecules through the extracellular matrix is hindered by narrow space, high tortuosity, and potentially, by a variety of physicochemical interactions [2]. We also want to caution that this PK profile might have been affected by the anesthesia state resulting in a higher CSF flow [1,31] and/or our ex vivo analysis. It has been shown that brain perfusion and fixation can cause the perivascular spaces to collapse and significantly reduce their size compared to their natural state in vivo, resulting in the release of the material into the parenchyma [32]. However, we believe our observation is not an ex vivo procedural artifact since the uptake of the antibody by perivenular spaces could not have occurred without prior transport from the periarteriolar spaces into the interstitial fluid of the parenchyma while the animal was still alive. In addition, the drug could not have appeared in the venules as a result of CSF bulk clearance since the antibody is observed in the perivenular spaces and not in the vasculature.

The observed time- and dose- dependency in our IV delivery study is in line with previous reports of systemically administered antibodies. Lee and Tannock reported the distribution of cetuximab and trastuzumab in tumor xenografts to be both time- and dose- dependent after systemic injection. These observations were not made in the healthy brain tissue, as was the case in our study, but rather in relation to distance from blood vessels and regions of hypoxia in the tumor [33]. Later on, two review papers by Lucas et al. mentioned time and dose as factors affecting the pharmacokinetic disposition of mAbs and ADCs within tumors [34,35]. In another study, a BBB penetrating bispecific antibody of 210 kDa in size displayed time- dependent brain concentrations, expressed as percent of injected dose per gram tissue (%ID/g). The brain exposure was higher within the first 8 h post IV injection, followed by a net elimination up to 24 h [36]. This observation matches the reports on time- and dose- dependent changes in antibody turnover and clearance as a function of the half-life [37,38].

Our preliminary study of target engagement with ABT-806 and ABT-414 in ICM implanted tumors shows that both the mAb and ADC can bind to the tumor cells though intra-CSF administration. Target engagement is achieved by the binding of these compounds to the epitope of interest on the human tumor cells. Incomplete ABT-806 penetration into the tumor cluster was observed within 30 min of injection. This raises the question as to whether the kinetic of transport within the tumor was diffusion-limited or target engagement-limited. We think that the answer is a mix of both: we did observe higher antibody penetration into the tumor core by increasing the circulation time to 1 h and 2 h. We also demonstrated that a 1:1 molar mixture of ABT-806 with ABT-414 increased the occupancy of the peripheral receptors by the parent antibody, leading to increased ADC penetration in the GBM cluster core. The latter is consistent with the receptor saturation hypothesis previously reported elsewhere [39].

Our toxicology study with ICM administration of ABT-414 indicates no microscopic findings in tissues exposed to the CSF flow, i.e., the brain, spinal cord, and eyes. The minimal liver findings in one rat at 1 mg/kg are consistent with limited ABT-414 systemic exposure that matches the exit pathway of the CSF circulation through the venous system [2]. The objective of the toxicology study was primarily to evaluate potential central nervous system (CNS) effects related to ICM delivery and associated distribution in the cerebral parenchyma. We did not intend to fully characterize the systemic toxicity of ABT-414 that has been comprehensively evaluated after IV administration in monkeys and mice. Mitotic arrest with secondary apoptotic cell death has been reported with microtubule inhibitors such as auristatin and maytansinoid derivatives in a diverse set of tissues, including the liver [40,41]. Minimal to mild mitotic arrest in the absence of associated tissue structural alteration is usually considered non-adverse. In the toxicology study reported here, increased mitoses/mitotic arrest was observed only in the liver (sinusoidal cells and hepatocytes) in a single rat dosed with ABT-414 at 1 mg/kg, indicating limited systemic exposure to ABT-414. In addition, the mitotic arrest was not associated with parenchymal injury and this minimal liver finding was non adverse. Future toxicology studies should include clinical pathology parameters such as liver enzymes levels to better correlate with our microscopic findings. Collecting toxicokinetic data exploiting bioanalytical measurements from the plasma would also help further evaluate the systemic exposure achieved after ICM delivery.

The ICM cannulation procedures used throughout this study provide a relatively simple and safe pathway to access the CSF circulation in rodents. Future studies in rodents will be conducted using ICV cannulation. This is because, unlike ICM delivery, ICV is translatable to humans.

Except for patients with head and neck injuries, ICM delivery via a suboccipital puncture is not seen as routinely translatable to clinical practice due to unacceptable procedural risks of serious, or fatal, complications. These can include inadvertent injury to vascular structures and brainstem damage. An alternate ICM route has been reported requiring the adaptation of intravascular microcatheter, which can be safely navigated intrathecally under fluoroscopic guidance. This approach to ICM delivery has been used to deliver viral vectors to the brain. Despite its promise, this ICM technique has not yet reached wide adoption [42].

Two approaches to intra-CSF drug delivery are currently considered acceptable in humans: Intrathecal (IT) or ICV delivery. With CSF “near stagnant” in the lumbar area, IT delivery requires pumping a higher initial dose to reach the SAS at therapeutic levels, a precondition to sustained penetration via the microcirculation system. In contrast, ICV delivery leverages brain physiology by infusing the drug proximal to the site of CSF production, thereby allowing the drug to go with the outward flow of the CSF in the direction of the SAS [2]. Despite its invasive nature, ICV delivery in humans is gaining more acceptance, for the treatment of acute and rapidly progressing conditions. A state of the art, fully implantable ICV system is currently used in a clinical trial to treat refractory epilepsy [43]. A comprehensive review of the safety of ICV delivery by Cohen-Pfeffer et al. can be found here [44].

## 5. Conclusions and Future Directions

This biodistribution data combined with preliminary evidence of no toxicity raise the hope that CSF delivery might be a viable route to expose diseased brain tissues to therapeutic mAbs or ADCs. This is important because the low permeability of the BBB to mAbs renders promising therapeutics ineffective or toxic when administered systemically, as observed in past clinical trials [11,13,14]. This was quite apparent in our study of IV delivered ABT-806, where trace amounts of antibody were detected in the brain tissue.

ABT-806 and ABT-414 were used as model molecules to determine whether intra-CSF delivery holds promise for the treatment of large regions of the brain. Their choice by no means implies that they are currently viewed as lead compounds for the treatment of GBM. It is too early to conclude that therapeutic doses can be achieved through this route of administration. Nonetheless this proof-of-concept study has intriguing ramifications for the future of drug delivery in both neuro-oncology and neuroscience.

While the preliminary PK profile obtained via image analysis provides useful insight on the rate of penetration and clearance of unbound molecules, it remains only semi-quantitative. Fully quantitative PK studies are underway by ex vivo analysis of brain homogenates as well as in vivo sampling of brain tissues using microdialysis after intra-CSF administration. Should PK studies confirm that a therapeutic dose is achievable with ABT-414, we may proceed to verify efficacy against cortically implanted GBM models.

Although commonly used in rodent studies, ICM delivery would not be suitable in humans for safety reasons. Our future preclinical studies will therefore utilize the ICV route of administration. A review of suppliers has revealed that ICV technology for human use has become safer and easier to use in recent years. While systemic delivery is clearly preferable over intra-CSF delivery, the desired outcome of this research would be a future where ICV delivery would enable therapies that would otherwise be prevented or hampered by side effects when administered systemically.

If the ability of achieving deep penetration of IgG-size biologics throughout the entire brain is confirmed in higher order species, the strategy of circumventing the BBB via CSF microcirculation would also be preferred over localized drug delivery modalities such as focused ultrasounds or intraparenchymal injections, especially when diseased tissues are present throughout large regions of the brain.

## Figures and Tables

**Table 1:** List of fluorescently labeled antibodies administered in vivo.

Antibody	Specifications
**ABT-806**	
Type	Parental Antibody—IgG1
Toxin Conjugate	None
FL Dye Conjugate	Alexa Fluor^®^ 555 NHS Ester (Thermo Fisher Scientific—#A20009)
FL Dye Excitation/Emission Wavelength	555/572 nm
FL Dye Extinction Coefficient	155,000 cm^−1^ M^−1^
FL Dye Molecular Weight	981 g/mol
**ABT-414**	
Type	Antibody-Drug Conjugate of ABT-806—IgG1
Toxin Conjugate	Monomethyl Auristatin F (MMAF)
FL Dye Conjugate	Atto 488 NHS ester (Sigma-Aldrich—#41698)
FL Dye Excitation/Emission Wavelength	500/520 nm
FL Dye Extinction Coefficient	90,000 cm^−1^ M^−1^
FL Dye Molecular Weight	1250 g/mol

**Table 2:** Meso Scale Discovery (MSD)-based ligand-binding assay for pharmacokinetics (PK) analysis.

**Plates**
MSD std bind streptavidin plates: MA6000 96 Plate (MSD cat# L11SA-1)
2 mL 96 well polypropylene plates
**Buffers**
Capture Dilution Buffer: Assay Buffer
Blocking Buffer: 3% MSD Blocker A (MSD cat# R93AA-1) in 1× Phosphate Buffered Saline (PBS)
Wash Buffer: 1× PBS with 0.05% Tween-20 (Obtained from Media Lab)
Standard Dilution Buffer: Assay Buffer
Sample Dilution Buffer for the first and subsequent dilutions: Assay Buffer
Detection Dilution Buffer: Assay Buffer
Assay buffer: 1% MSD Blocker A, in 1× Tris-Buffered Saline (TTBS with 0.02% Tween-20)
Read Buffer: MSD GOLD READ BUFFER A (MSD cat# R92TG-2) is provided at the working concentration and is used at this supplied concentration without any additional dilution.
**Equipment**
Tecan EVO
MSD QuickPlex 120 Reader
Biotek Stacker Plate Washer
VWR Microplate Shaker
**Reagents**
Goat anti Human IgG Fc Biotin
Goat anti-human IgG Sulfo TAG = MSD cat # R32AJ-1
**Standard Range: In Duplicate**
- 25–0.102 ng/mL with 2.5 serial dilution (made with assay buffer) - Predilute test article 1:200 in PBS by doing a 1:20 dilution (3 µL of stock/57 µL of PBS then further dilute 1:10 (5 µL of previous 1:20/45 µL of PBS) - Add 6.47 µL of the final predilution into 143.5 µL of assay buffer to make a 2.5 µg/mL Standard - Serial dilute top standard 1:2.5 in assay buffer 7 times
**Quality Controls: In Duplicate (made with assay buffer)**
- High = 1.75 µg/mL (4.53 µL of final predilution/145.5 µL of assay buffer) - Medium = 0.350 µg/mL (1:5 dilutions from High control—20 µL high/80 µL of assay buffer) - Intermediate = 0.070 µg/mL (1:5 dilutions from Medium control—20 µL high/80 µL of assay buffer) - Low = 0.014 µg/mL (1:5 dilutions from Intermediate control—20 µL high/80 µL of assay buffer)

**Table 3:** Immunohistochemistry assay for toxicology study.

Antibody	Vascular Endothelial Growth Factor Receptor 2 (VEGFR2)
**Primary antibody**	
Source	Cell Signaling
Catalog number	9698
Lot number	4
Concentration	59 μg/mL
Dilution	0.6 μg/mL (~1:100)
Incubation time	60 min
Negative control	Rabbit IgG
Source	Abcam
Catalog number	ab172730
Tissues used as positive controls	Liver
**Secondary antibody**	
Source	BOND Polymer Refine Detection
Catalog number	DS9800
Lot number	69657
Concentration	N/A
Dilution	RTU
Incubation time	8 min
Antigen Retrieval	ER2; pH 9; 40 min on LEICA BOND RX automated stainer
Chromogen	DAB

DAB: 3,3′-Diaminobenzidine tetrahydrochloride hydrate; RTU: Ready to Use.

## Data Availability

The data that support the findings of this study are available upon reasonable request.
